# Current and Future Incidence and Costs of Osteoporosis-Related Fractures in The Netherlands: Combining Claims Data with BMD Measurements

**DOI:** 10.1007/s00223-015-0089-z

**Published:** 2016-01-09

**Authors:** Freek J. B. Lötters, Joop P. van den Bergh, Frank de Vries, Maureen P. M. H. Rutten-van Mölken

**Affiliations:** Institute of Medical Technology Assessment, Erasmus University, P.O.-Box 1738, 3000 DR Rotterdam, The Netherlands; Maastricht University Medical Center+, Maastricht, The Netherlands; VieCuri Medical Center Noord Limburg, Venlo, The Netherlands; Department of Clinical Pharmacy & Toxicology, Maastricht University Medical Center+, Maastricht, The Netherlands; MRC Epidemiology Lifecourse Unit, Southampton General Hospital, Southampton, UK; Utrecht Institute for Pharmaceutical Sciences, Utrecht, The Netherlands

**Keywords:** Incidence, Costs, Osteoporosis, Fractures, Future projections

## Abstract

This study aims to estimate the incidence and costs of osteoporosis-related fractures in The Netherlands in 2010 and project them to 2030. The incidence and costs of five different types of fractures (spine, hip, upper extremity, lower extremity, wrist/distal forearm, other) were derived from claims data of all Dutch healthcare insurers. Given that fracture-codes in claims data do not indicate whether fractures are related to osteoporosis, we used a large dataset with DXA measurements to attribute fractures to osteoporosis. Future projections used four scenarios: (1) demographic, (2) demographic + annual trend in incidence rates, (3) demographic + annual trend in incidence rates + annual trend in costs, and (4) treatment. Of all registered fractures, 32 % was attributed to osteoporosis (36 % in women and 21 % in men). Over time (2010–2030) the increase in incidence of osteoporosis-related fractures was estimated to be 40 % (scenario 1); for the hip 60–79 % (scenario 1–2). In 2010, approximately €200 million was spent on treatment of osteoporosis-related fractures, most on fractures of the hip followed by wrist/distal forearm. In both men and women, the excess costs due to osteoporosis-related fractures were highest for hip fractures (€11,000–€13,000 per person), followed by spine fractures (€6000–€7000).The costs for osteoporosis-related fractures were projected to increase with 50 % from 2010 to 2030 (scenario 1); for the hip 60–148 % (scenario 1–3). Pharmacotherapeutic prevention can lead to cost-savings of €377 million in 2030 (scenario 1 and 4 combined). The projected increase in incidence and costs of osteoporosis-related fractures calls for a wider use of prevention and treatment.

**Electronic supplementary material:**

The online version of this article (doi:10.1007/s00223-015-0089-z) contains supplementary material, which is available to authorized users.

## Introduction

The prevalence of osteoporosis in The Netherlands is estimated at 1.9 per 1000 for men and 16.1 per 1000 for women and it increases strongly with age [1]. Osteoporosis increases the risk of fractures [2]. An osteoporosis-related fracture at higher age substantially impairs the quality of life and increases the costs of healthcare [3].

Important risk factors and prognostic factors of osteoporosis and osteoporosis-related fractures include age, sex, bone mineral density, spine fracture in the past, a recent none-spine fracture, a non-recent fracture above the age of 50, and a low body weight [2]. Although the guidelines strongly recommend diagnosing osteoporosis by dual-energy X-ray absorptiometry (DXA) measurements in patients with clinical risk factors, the implementation of this recommendation highly varies between settings [4]. As a result, the prevalence estimates are affected by considerable underrepresentation and underdiagnosis and only a part of the target group will be treated for osteoporosis [5].

Treatment of osteoporosis consists of lifestyle recommendations, vitamin D supplementation and specific anti-osteoporosis medication [5]. Bisphosphonates are the most widely prescribed anti-osteoporosis medication.

However, therapy adherence is low. Low adherence not only has medical implications, but also economical because the treatment of a (subsequent) fracture is rather costly [2, 3] and a fracture may cause permanent disability associated with high healthcare use.

With an ageing population, and a growing proportion of people of 80 years or older, the societal burden of osteoporosis-related fractures is deemed to be increasing [6]. However, we do not know to what extent, because there is a lack of detailed information on the gender and age-specific incidence and healthcare costs of osteoporosis-related fractures needed to enable future projections [2]. Previous studies in The Netherlands have reported the costs of fractures without attributing a proportion of these costs to osteoporosis [7]. This study aims at giving more insight in the number of osteoporosis-related fractures and the associated healthcare costs in The Netherlands. Moreover, projections will be made of the number of osteoporosis-related fractures and the related costs in the near future. We have specifically investigated the incidence of fractures among patients aged 50 years or older, the proportion of these fractures that is due to osteoporosis, the healthcare costs due to osteoporosis-related fractures and the projections of osteoporosis-related fractures and related costs over time between 2010 and 2030.

## Methods

### Overall Incidence of Fractures

#### Data Source

Data on the incidence of fractures were obtained from the VEKTIS database. This health insurance claims database includes the healthcare expenditures of 99 % of the 16.5 million Dutch citizens. Since 2006, the Dutch citizens have a single compulsory healthcare insurance for the basic benefit package that includes primary care, outpatient hospital care, inpatient hospital care and medications; they may have supplementary insurance on top of that. Insurers are free to contract providers based on negotiations on price and quality and patients choose the provider they prefer. Health insurers are obliged to accept all new applicants for the basis benefit package and they are not allowed to differentiate the premiums of the basic benefit package according to risk profile. The government controls the quality, accessibility and affordability of health care. Inpatient and outpatient hospital care is paid by the insurers through an elaborate diagnosis-related groups (DRG) system. In essence, hospitals receive a fixed payment per Diagnosis-Treatment-Combination (DBC). This DBC system was implemented in The Netherlands in 2005. Currently, nationwide DBCs are registered in the VEKTIS database. The exact services provided in a particular DBC are not registered separately. Due to a steep learning curve in the registration of DBCs in the early years after the introduction and changes in the DBC registration system, VEKTIS data before 2009 are less valid and data from 2012 onwards were incomplete on the date of data extraction for this study (i.e. February 05, 2014). Therefore, we used data from the VEKTIS database for the years 2009, 2010, and 2011.

#### Study Population

To estimate the incidence rates of fractures, we selected from the VEKTIS database all subjects aged 50 years or over, with at least one DBC code for a fracture as recorded by orthopaedic specialists or general surgeons. Fracture DBC codes were categorized according to the following fracture sites: upper extremity, wrist/distal forearm, spine, lower extremity, hip, and other fractures. The list of codes is provided in the Online Appendix. The VEKTIS data were grouped by gender and 5-year age categories (i.e. 51–55, 56–60, 61–65, 66–70, 71–75, 76–80, 81–85 years, and older than 85 years). Absolute and relative incidence (defined as the number per 100,000 person years) of fractures per year were calculated for each age category in men and women. To calculate the relative incidence, we used the size of the population, as reported by Statistics Netherlands (CBS) [8].

### Incidence of Osteoporosis-Related Fractures

In order to attribute fractures to osteoporosis, we used a dataset of the VieCuri Medical Center, an expert center on metabolic bone disease, in the southern part of The Netherlands. This dataset contained bone-mineral-density (BMD) measures (T-scores) of people 50 years or older who visited the hospital due to a fracture between 2009 and 2011 [9, 10]. In line with the WHO, we defined osteoporosis as a BMD of 2.5 or more standard deviations below that of a healthy 30-year old adult (i.e. a T-score of ≤ −2.5 SD) as measured by DXA at the lumbar spine, femoral neck or total hip [11]. All fractures in patients with a T-score of ≤−2.5 SD were defined as being related to osteoporosis [5, 11]. To calculate the total number of osteoporosis-related fractures, the percentage of fractures with a T-score ≤ −2.5 SD was applied to the total number of fractures obtained from the VEKTIS data. This was done separately for each fracture site (i.e. hip, spine, wrist/distal forearm, upper extremity, lower extremity, and other).

### Healthcare Costs

Healthcare costs in the years 2009–2011 were also obtained from VEKTIS data. These costs included all inpatient and outpatient hospital costs (per DBC), costs of physical therapy, occupational therapy, general practitioner contacts and medication. We obtained the mean gender and age-specific healthcare costs of people with a fracture of a particular type and compared them to the mean gender and age-specific healthcare costs of people without a fracture. This way, we calculated the excess healthcare costs of a patient with a fracture compared to a patient without a fracture, by gender and age category. To estimate the total healthcare costs due to osteoporosis-related fractures, we multiplied the absolute incidence of osteoporosis-related fractures in each gender, age, and fracture category with the mean excess healthcare cost in the same category. Costs were expressed in Euros (€).

### Future Projections and Scenarios

Using the incidence and costs of osteoporosis-related fractures in 2010 as a reference four future scenarios are presented that project the incidence and costs of osteoporosis-related fractures from 2010 to 2030:

1. A demographic scenario for incidence and costs for all osteoporosis-related fractures

In this scenario, the gender and age-specific incidence rate per 100,000 in 2010 was multiplied by the number of citizens in each gender and age class that is projected by the CBS for the years 2015, 2020, 2025, and 2030 [8].

2. A demographic scenario for incidence that additionally included the annual trend in the incidence of hip fractures that was observed between 2000 and 2010.

The annual trend in incidence of hip fractures was calculated separately for each 5-year age- and gender category using data from the National Medical Register of Dutch hospitals (LMR) from 2000 to 2010 [12]. We had to use data from the LMR to estimate this trend because the VEKTIS data covered only 3 years, which is insufficient to estimate a trend. The LMR is a national register of hospital admissions, including admissions due to fractures. However, the LMR is less representative than the VEKTIS data because the number of missing records in the LMR has increased since 2005, probably because of the stepwise introduction of the DBC system in hospitals [13]. Comparison of the number of fractures in the LMR data for 2009, 2010, and 2011 with the number of fractures in the VEKTIS data revealed that only the number of hip fractures was similar. All other fracture types seem systematically underreported in the LMR data compared to the VEKTIS data. We therefore decided to estimate only the trend in hip fractures.

3. A demographic scenario for costs of osteoporosis-related hip fractures that additionally included the annual trend in the incidence of hip fractures and the annual trend in healthcare costs.

The annual increase in healthcare costs is obtained from the Dutch Healthcare Authority (NZa). This increase was estimated to be 1.6 % per year over the period 2006–2010. This is the increase in prices after correction for the increase in the amount of healthcare resource utilization [14].

4. A treatment scenario for costs of osteoporosis-related *hip* fractures. We applied a treatment-induced relative risk reduction for osteoporosis-related fractures to the results from scenario 3.

The relative risk reductions of interventions preventing osteoporosis-related fractures were obtained from the Cochrane Review database [15]. We used the estimate for the most prominent intervention applied in The Netherlands, i.e. alendronate [5, 16].With the obtained risk reduction an estimate could be given of future cost savings due to treatment.

## Results

Over the years 2009–2011, the VEKTIS database contained on average 5,616,439 people of 50 years or older per year. Over these years an annual average of 114,116 patients with a fracture was identified, divided into 15 % hip fractures, 6 % fractures of the spine, 22 % distal forearm/wrist fractures, 26 % upper extremity fractures, 24 % lower extremity fractures, and 7 % other fractures.

### Incidence Osteoporosis-Related Fractures

From the VieCuri Medical Center, DXA measurements of 1984 patients older than 50 years (529 male, 1455 female) who presented themselves with a recent fracture were available. Of these patient 8.6 % had a hip fracture, 8.9 % a fracture of the spine, 17.3 % a distal forearm/wrist fracture, 33.6 % a fracture located at the upper extremity, 22.5 % a fracture located at the lower extremity, and 9.2 % another kind of fracture. Table 1 shows the percentage of fractures attributed to osteoporosis (i.e. fractures in patients with a T-score of −2.5 or less at lumbar spine, femoral neck or total hip).

**Table 1:** Number and percentage of fractures with osteoporosis (T-score ≤ −2.5 at lumbar spine, femoral neck or total hip) by gender and fracture type

	Men		Women	
	*n* (*N*)	%	*n* (*N*)	%
Spine	18 (54)	33.3	65 (123)	52.8
Hip	22 (60)	36.7	60 (108)	55.6
Upper extremity	24 (137)	17.5	180 (529)	34.0
wrist/distal forearm	9 (58)	15.5	100 (285)	35.1
Lower extremity	26 (146)	17.8	77 (300)	25.7
Other	12 (73)	16.4	44 (109)	40.4
Total	111 (528)	21.0	526 (1454)	36.2

*n* = Number of fractures attributed to osteoporosis based on T-score of DXA; *N* = total number of fractures

Overall, 32 % of the fractures could be attributed to osteoporosis. This percentage was considerably higher for women than for men, i.e. 36 % versus 21 %, respectively. More than 50 % of hip fractures and spine fractures in women were attributed to osteoporosis.

In Table 2 the absolute and relative incidence of all fractures and osteoporosis-related fractures are presented for 2010. To obtain the number of osteoporosis-related fractures the gender and age-specific number of fractures of each type were multiplied by the gender and fracture-specific percentages as summarized in Table 1. In Tables A1, A2, and A3 of the Online Source the incidence of fractures by 5-year age- and gender class is given.

**Table 2:** Incidence of all fractures and osteoporosis-related fractures, by gender and fracture type, in 2010

	Fracture type	All fractures	Osteoporosis-related fractures (absolute)	Osteoporosis-related fractures (/100,000)
Male	Spine	2285	761	27
	Hip	5219	1915	69
	Upper extremity	9924	1737	62
	Wrist/distal forearm	5227	810	29
	Lower extremity	8461	1506	54
	Other	3094	507	18
	Total	34,210	7236	260
Female	Spine	4459	2354	76
	Hip	12,543	6974	226
	Upper extremity	20,742	7052	228
	Wrist/distal forearm	22,120	7764	251
	Lower extremity	20,094	5164	167
	Other	5251	2121	69
	Total	85,209	31,429	1018
Male and Female	Spine	6744	3115	53
	Hip	17,762	8889	151
	Upper extremity	30,666	8789	150
	Wrist/distal forearm	27,347	8574	146
	Lower extremity	28,555	6670	114
	Other	8345	2629	45
	Total	119,419	38,666	659

When looking at all fractures, regardless of their relationship to osteoporosis, the number of women with a fracture was more than twice the number of men. When looking at osteoporosis-related fractures, the incidence was more than four times higher in women than in men. In men, osteoporosis-related fractures of the hip were most frequent, whereas in women osteoporosis-related fractures of the wrist/distal forearm were most frequent.

### Projections of Incidence of Osteoporosis-Related Fractures

In the demographic scenario, the age- and gender-specific incidence rates per 100,000 in 2010 were applied to the projected numbers of people in the population. Due to ageing of the population, the total number of osteoporosis-related fractures increased from 38,666 in 2010 to 53,993 in 2030. The increase in incidence of osteoporosis-related fractures from 2010 to 2030 was 40 %; 48 % in men and 38 % in women.

**Fig. 1:**
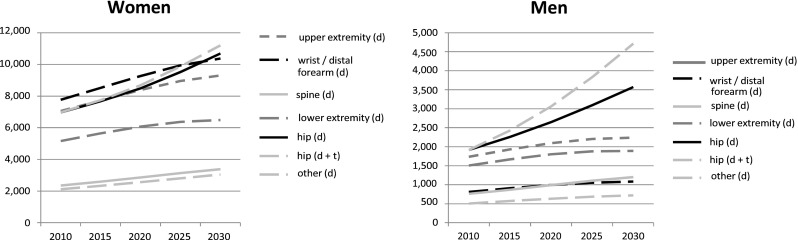
Projected number of osteoporosis-related fractures over time by type of fracture (in women and men); demographic scenario (d) and demographic trend (d + t) in incidence scenario (only for osteoporosis-related hip fractures)

In Fig. 1, the demographic projections from 2010 to 2030 are presented by type of osteoporosis-related fracture.

In both men and women, osteoporosis-related fractures of the hip showed the largest increase in incidence. In women, this increase was 53 % whereas in men this increase was 86 %.

In Table A4 of the Online Source, the annual trend in incidence of hip fractures per 100,000 persons from 2000 until 2010 is presented. When we added this trend to the demographic scenario, the steeper increase in osteoporosis-related hip fractures in men as compared to women was even more pronounced. In men, the number of osteoporosis-related hip fractures in 2030 was 32 % higher in the demographic plus incidence trend scenario compared with the demographic scenario. In women, the difference between the two scenarios was only 5 %.

### Costs of Osteoporosis-Related Fractures

Table 3 shows that the annual healthcare costs for osteoporosis-related fractures in 2010 were almost 200 million Euros a year; 77 % of these costs were due to osteoporosis-related fractures in women. In both men and women, the costs of osteoporosis-related hip fractures were the largest; 56 % of total costs in men and 52 % of total costs in women. Approximately 80 % of all healthcare costs incurred by patients with an osteoporosis-related hip fracture could be attributed to treatment and care directly related to this fracture. For osteoporosis-related spine fractures, this percentage was approximately 70 %, whereas for fractures of the wrist/distal forearm it was only 33 %.

**Table 3:** Annual healthcare costs for osteoporosis-related fractures in 2010

	Fracture type	Mean healthcare costs for a person with a fracture (fracture and non- fracture related)*	Mean healthcare costs for a person without a fracture (non-fracture related)*	%cost related to fractures*	Mean healthcare costs due to osteoporosis-related fracture per person*	Total healthcare costs due to osteoporosis-related fractures in The Netherlands**
Men	Spine	€10,786.03	€3578.88	67	€7207.15	€5,483,953.08
	Hip	€16,708.45	€4098.85	75	€12,609.60	€24,152,080.40
	Upper extremity	€5463.60	€2862.24	47	€2601.35	€4,517,772.00
	Wrist/distal forearm	€4751,15	€3005.04	37	€1746.11	€1,414,670.73
	Lower extremity	€6301.98	€2793.01	56	€3508.97	€5,284,706.88
	Other	€7832.67	€3167.51	60	€4665.17	€2,367,180.95
	Total					€43,220,364.04
Women	Spine	€8834.83	€3258.86	63	€5575.97	€13,127,801.27
	Hip	€14,545.30	€3534.72	76	€11,010.57	€76,786,727.38
	Upper extremity	€5610.96	€2903.45	48	€2707.51	€19,094,139.49
	Wrist/distal forearm	€4426.40	€2941.75	34	€1484.65	€11,527,023.52
	Lower extremity	€6034.66	€2718.38	55	€3316.27	€17,125,767.07
	Other	€7603.95	€3257.15	58	€4346.80	€9,221,309.75
	Total					€146,882,768.48
Men and Women	Spine	€9495.93	€3367.29	64	€6128.65	€18,611,754.35
	Hip	€15,180.89	€3700.48	76	€11,480.41	€100,938,807.77
	Upper extremity	€5563.27	€2890.11	48	€2673.16	€23,611,911.49
	Wrist/distal forearm	€4488.47	€2953.85	34	€1534.63	€12,941,694.25
	Lower extremity	€6113.87	€2740.50	55	€3373.37	€22,410,473.95
	Other	€7688.75	€3223.91	58	€4464.84	€11,588,490.70
	Total					€190,103,132.51

* Weighted average over the 5-year age classes within each fracture type

** Determined by the incidence figures presented in Table 2

In both women and men, the healthcare costs due to osteoporosis-related hip fractures (approximately €11,000–€13,000 per person) exceeded those of other osteoporosis-related fractures substantially. The healthcare costs of osteoporosis-related spine fractures were the second highest category with approximately €5500–€7000 per fracture.

**Fig. 2:**
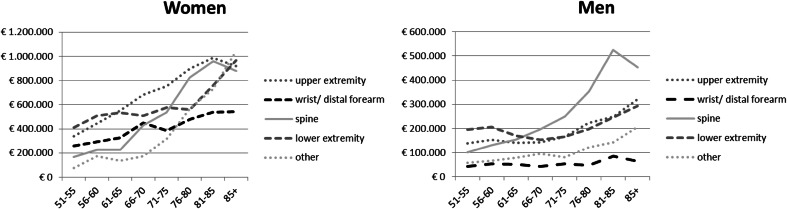
Costs of osteoporosis-related fractures in 2010 per 100,000 person years in each age class

In Fig. 2, the costs of osteoporosis-related fractures in 2010 are presented per 100,000 person years in each age class. In both men and women costs of osteoporosis-related fractures increased with age. In men, costs at age 85+ were 1.5 times as high as the costs at age 51–55. In women, costs at age 85+ were 2.5 times as high as the costs at age 51–55. In both men and women, the difference between the youngest and oldest age category was highest for other fractures and spine fractures.

### Projections of Costs of Osteoporosis-Related Fractures

Due to ageing of the population, the overall increase in costs of osteoporosis-related fractures between 2010 and 2030 were 50 %. For women this increase was 45 %, whereas for men it was 66 % (demographic projection). After osteoporosis-related hip fractures, with a cost increase of 52 and 84 % for women and men, respectively, the costs of osteoporosis-related spine and other fractures increased most (ranging from 43 to 56 %).

**Fig. 3:**
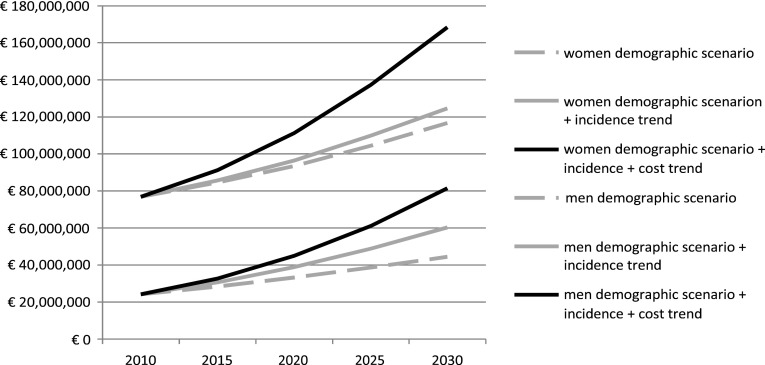
Estimated costs of osteoporosis-related hip fractures till 2030 (in men and women); demographic scenario, demographic + incidence trend scenario, and demographic + incidence trend + costs trend scenario

Figure 3 shows the change in costs of osteoporosis-related hip fractures over time for the demographic scenario, the demographic plus trend in incidence scenario, and the demographic plus trend in incidence and trend in costs scenario. For the first scenario, the increase in healthcare costs over time was 52 % in women and 84 % in men.

For the demographic scenario including the incidence trend, these percentages were 62 and 157 %, respectively. When the trend in costs was added (i.e. the upper line), costs increased with 119 % for women and 237 % for men between 2010 and 2030.

### Cost savings by Intervention

The bisphosphonate alendronate is the most frequently prescribed medication for osteoporosis in The Netherlands [17]. A Cochrane review [18] showed that the use of alendronate (10 mg/day) reduced the risk of osteoporosis-related fractures by 16–45 %, yielding relative risks of 0.60 (95 % CI 0.40–0.92) for hip fracture, 0.55 (95 % CI 0.45–0.67) for spine fracture, and 0.84 (95 % CI 0.74–0.94) for non-spine fracture. These relative risks were used as an indicator of the potential effect size of preventive interventions.

Applying the above-mentioned effect-sizes to the numbers presented in Fig. 3, the cumulative cost savings in the period from 2010 until 2030 were estimated to be 377 million Euro (280 million for women and 97 million for men). Costs due to osteoporosis-related hip fractures could be decreased with 258 million, osteoporosis-related spine fractures with 52 million and other osteoporosis-related fractures with 67 million.

## Discussion

This study showed that of all registered fractures in The Netherlands, 32 % (36 % in women and 21 % in men) could be attributed to osteoporosis. This resulted in an incidence of osteoporosis-related fractures of 1018 per 100,000 for women and 260 per 100,000 for men in 2010. Between 2010 and 2030, the total number of osteoporosis-related fractures was projected to increase with 40 % (demographic scenario). Hip fractures were the most frequent type of osteoporosis-related fractures, with a projected increase that was higher in men (86 %) than in women (53 %) (demographic scenario).

In 2010, Dutch healthcare insurers spent approximately €200 million on treatment of osteoporosis-related fractures. 55 % of these costs were due to osteoporosis-related hip fractures. The increase in costs with age was steepest for spine fractures, where the costs in the oldest female age group were 2.5 times as high as in the youngest female age group. The costs of osteoporosis-related fractures were projected to increase with 50 % between 2010 and 2030 (demographic scenario). The increase in costs over time was larger for men than for women, 66 and 45 %, respectively. Taking into account the annual trend in incidence and the trend in healthcare costs in general, the costs for osteoporosis-related hip fractures in 2030 were projected to be €168 million for women and €81 million for men.

For the demographic projections, we used the gender- and age-specific incidence rate of 2010 and kept it constant over time. Hence, the increase in the number of fractures is due to ageing of the population. The estimated trend in incidence rate that we put on top of the demographic projection was based on the yearly change in incidence rate of hip fractures between 2000 and 2010 within each age category. Hence, this trend is not due to ageing but, for example, due to an increase in the prevalence and/or severity of osteoporosis.

In our study, we defined an osteoporosis-related fracture as a fracture that occurred while the DXA measurement afterwards showed a T-score of ≤ −2.5. Another approach often used in studies is to consider all fractures from low energy trauma as being related to osteoporosis [19]. The merit of this approach is that is recognizes that many fragility fractures occur at less extreme BMD values. However, the definition of a low energy fall as a fall from a standing height or less, or a trauma that in a healthy individual would not give rise to fracture, is hard to measure objectively. Hence, the assessment of BMD is most frequently used to define an osteoporosis-related fracture. Related to the discussion about the definition of an osteoporosis-related fracture is the discussion about diagnosis of a spinal fracture. A spinal fracture can either be clinically diagnosed (without X-ray) or morphometrically (diagnosed with X-ray) [20, 21]. In this study, no distinction could be made between these diagnosis types. However, based on clinical practice, most of the vertebral fractures registered by orthopaedic and trauma surgeons in The Netherlands will be clinically diagnosed fractures, since systematic evaluation for morphometric fractures is not the current practice. Furthermore, it is known that especially morphometric spinal fractures are often undetected and therefore underdiagnosed [20, 21]. This implicates that in our study the cost for treatment of spinal fractures is probably underestimated.

A major strength of our study is that we had almost complete claims data on fractures, because the VEKTIS database covers 99 % of the 16.5 million Dutch citizens. We could combine this with DXS scan data to attribute a proportion of these fractures to osteoporosis.

The DXS data were obtained from the VieCuri Medical Center, an expert center on metabolic bone disorders, in the southern part of The Netherlands. This dataset contained approximately 70 % of all fracture patients, i.e. those that were seen at the fracture liaison service (FLS) and agreed to be examined for osteoporosis with a DXA scan. Approximately 30 % of patients were not able or willing to participate in the FLS. These “non- attenders” appeared to be significantly older and had more hip fractures than the attenders [22]. This might have induced a selection bias, which has probably led to an underestimation of osteoporosis-related fractures. The DXS data of the VieCuri Medical Center are part of a study among five large hospitals in The Netherlands that offer a FLS. Although differences between the patients included in the FLSs exist, from a clinical point of view, these differences were small, indicating that patient enrollment was quite similar between FLSs [4]. In this regard, the database on DXA measurements that we used can be considered representative for The Netherlands.

The VEKTIS claims database also has some limitations. It only contains the healthcare expenditures reimbursed by the health insurance companies. Long-term residential care costs or costs made for home care were not included. These costs may be substantial. A Dutch cost of illness study that uses a top-down approach to allocate costs to diseases [7] reported that 54 % of the costs of hip fractures were due to costs of care given in a hospital and 37 % due to long-term residential care. We estimated that the costs of hospital care for all osteoporosis-related fractures were €151 million. Applying the percentages from the Dutch Costs of Illness study to the €151 million hospital care cost in our study (37 %/54 %*151 million) an additional €103 million needs to be added for long-term residential care. This would increase our total cost estimate from €190 to €293 million for all osteoporosis-related fractures in 2010.

The VEKTIS database includes claims data covered by the obligatory basic insurance or the voluntary supplementary insurance. Physical therapy is partly reimbursed from the basic insurance but mostly from the supplementary insurance. Costs of physical therapy by patients without supplementary insurance will be paid by the patients themselves. These out-of-pocket costs are not included in our calculations. However, we are convinced that this accounts only for a small fraction of the total healthcare expenditure for osteoporosis-related fractures because approximately 90 % of the insured did have such a supplementary insurance in 2010 [23].

Furthermore, the use of DBC-data might induce underreporting when patients have multiple fractures. Generally, only one fracture DBC can be registered in the same period. This underreporting is probably less prominent for hip fractures than for the other clinical fractures.

There is a large variation in the incidence of osteoporosis-related fractures between countries. Age- and sex-specific risks of hip fracture differ more than 10-fold, even within Europe [2]. It is hypothesized that this is related to differences in socio-economic prosperity, which in turn may be related to low levels of physical activity. However, the frequency of osteoporosis-related fractures is rising in many countries, primarily due to increased longevity of the population. Our estimates of the future increase in costs were well in line with a large European study in which an increase in costs of osteoporosis-related fractures of 25 % were presented for all EU27 countries. For The Netherlands this European study projected an increase of 30 % until 2025 [2]. In our study, the overall increase in healthcare costs from 2010 until 2025 were 37 %. Remarkably, the increase in healthcare costs over time was greater for men than for women. This can probably be explained by a stronger increase in remaining life expectancy at age 65, because the difference between women and men in life expectancy at age 65 decreased from 3.2 years in 2010 to 2.0 years in 2030 [24]. This gender difference in the future increase in fractures was also seen in the aforementioned European study [2].

Using a bisphosphonate (alendronate) as medication to prevent fractures will save approximately €258 million for hip fractures, €52 million for spine fractures, and €67 million for other fractures in the period 2010–2030, assuming adequate medication persistence. Alendronate is recommended as one of the first treatment options for the primary prevention of osteoporosis-related fragility fractures [5, 25]. In The Netherlands, within the group of bisphosphonates, alendronate accounts for 57 % of the total number of defined daily doses that are dispensed [17]. However, adherence to bisphosphonates is a problem. Half of the patients diagnosed with osteoporosis do not adhere to the prescription after 1 year [2, 5], whereas bisphosphonates are prescribed mostly for 5 years [5]. This non-adherence increases the risk of a follow-up fracture.

In conclusion, with an incidence of 39,000 per year and concomitant healthcare costs of at least €200 million osteoporosis-related fractures are a considerable burden for Dutch society. Projections of incidence and healthcare costs over time indicate that this burden will increase by 40–50 % between 2010 and 2030. Future healthcare expenditures can be reduced by applying bisphosphonates in preventing the occurrence of osteoporosis-related fractures.

## Electronic supplementary material

Supplementary material 1 (DOCX 24 kb)
