# Supplementary material for: Current and Future Incidence and Costs of Osteoporosis-Related Fractures in The Netherlands: Combining Claims Data with BMD Measurements
Source: Calcif Tissue Int. 2016 Jan 9;98:235–43. doi: 10.1007/s00223-015-0089-z (PMC4746227; doi:10.1007/s00223-015-0089-z)
Supplement: Supplementary file 1 — Supplementary material 1 (DOCX 24 kb) [file 223_2015_89_MOESM1_ESM.docx]

**Online Source**

**Table A1. Incidence of all fractures in the Netherlands after age 50, by gender, fracture type and age category in 2010.**

|  |  | **All fractures** |  |  |  |  |  |  |  | |  |
| --- | --- | --- | --- | --- | --- | --- | --- | --- | --- | --- | --- |
|  | **Fracture type** | **51-55** | **56-60** | **61-65** | **66-70** | **71-75** | **76-80** | **81-85** | **85+** | **total** | |
| **Male** | spine | 244 | 297 | 342 | 272 | 298 | 346 | 283 | 203 | | 2,285 |
|  | hip | 264 | 370 | 492 | 504 | 611 | 889 | 956 | 1,133 | | 5,219 |
|  | upper extremity | 2,492 | 2,035 | 1,741 | 1,121 | 812 | 768 | 535 | 420 | | 9,924 |
|  | Wrist/ distal forearm | 415 | 479 | 482 | 449 | 582 | 794 | 840 | 1,210 | | 5,251 |
|  | lower extremity | 2,153 | 1,804 | 1,563 | 985 | 777 | 544 | 387 | 248 | | 8,461 |
|  | Other | 556 | 542 | 523 | 380 | 326 | 301 | 247 | 219 | | 3,094 |
|  | Total | 6,124 | 5527 | 5,143 | 3,711 | 3,406 | 3,642 | 3,248 | 3,433 | | 34,234 |
| **Female** | Spine | 295 | 387 | 432 | 538 | 607 | 765 | 730 | 705 | | 4,459 |
|  | Hip | 280 | 480 | 703 | 782 | 1,199 | 2,082 | 2,778 | 4,239 | | 12,543 |
|  | upper extremity | 2,775 | 3,122 | 3,466 | 2,655 | 2,472 | 2,396 | 1,985 | 1,871 | | 20,742 |
|  | Wrist/ distal forearm | 2,359 | 3,348 | 3,758 | 3,115 | 2,771 | 2,743 | 2,205 | 1,821 | | 22,120 |
|  | lower extremity | 3,685 | 3,815 | 3,583 | 2,593 | 2,032 | 1,651 | 1,313 | 1,422 | | 20,094 |
|  | Other | 415 | 479 | 482 | 449 | 582 | 794 | 840 | 1,210 | | 5,251 |
|  | Total | 9,809 | 11,631 | 12,424 | 10,132 | 9,663 | 10,431 | 9,851 | 11,268 | | 85,209 |
| **M&F** | Spine | 539 | 684 | 774 | 810 | 905 | 1,111 | 1,013 | 908 | | 6,744 |
|  | Hip | 544 | 850 | 1,195 | 1,286 | 1,810 | 2,971 | 3,734 | 5,372 | | 17,762 |
|  | upper extremity | 5,267 | 5,157 | 5,207 | 3,776 | 3,284 | 3,164 | 2,520 | 2,291 | | 30,666 |
|  | Wrist/ distal forearm | 2,774 | 3,827 | 4,240 | 3,564 | 3,353 | 3,537 | 3,045 | 3,031 | | 27,371 |
|  | lower extremity | 5,838 | 5,619 | 5,146 | 3,578 | 2,809 | 2,195 | 1,700 | 1,670 | | 28,555 |
|  | Other | 971 | 1,021 | 1,005 | 829 | 908 | 1,095 | 1,087 | 1,429 | | 8,345 |
|  | Total | 15,933 | 17,158 | 17,567 | 13,843 | 13,069 | 14,073 | 13,099 | 14,701 | | 119,443 |

24

**Table A2. Incidence of osteoporosis-related fractures, by gender (absolute), fracture type and age category, in 2010.**

|  |  | **Osteoporosis-related fractures (absolute)** | | | | | | | | | |
| --- | --- | --- | --- | --- | --- | --- | --- | --- | --- | --- | --- |
|  | **Fracture type** | **51-55** | **56-60** | **61-65** | **66-70** | **71-75** | **76-80** | **81-85** | **85+** | **total** | |
| **Male** | spine | 81 | 99 | 114 | 91 | 99 | 115 | 94 | 68 | | 761 |
|  | hip | 97 | 136 | 181 | 185 | 224 | 326 | 351 | 416 | | 1915 |
|  | upper extremity | 436 | 356 | 305 | 196 | 142 | 134 | 94 | 74 | | 1737 |
|  | Wrist/ distal forearm | 162 | 151 | 149 | 114 | 86 | 68 | 48 | 32 | | 810 |
|  | lower extremity | 383 | 321 | 278 | 175 | 138 | 97 | 69 | 44 | | 1506 |
|  | Other | 91 | 89 | 86 | 62 | 53 | 49 | 41 | 36 | | 507 |
|  | Total | 1250 | 1152 | 1113 | 824 | 744 | 790 | 696 | 669 | | 7237 |
| **Female** | Spine | 156 | 204 | 228 | 284 | 320 | 404 | 385 | 372 | | 2354 |
|  | Hip | 156 | 267 | 391 | 435 | 667 | 1158 | 1545 | 2357 | | 6974 |
|  | upper extremity | 944 | 1061 | 1178 | 903 | 840 | 815 | 675 | 636 | | 7052 |
|  | Wrist/ distal forearm | 828 | 1175 | 1319 | 1093 | 973 | 963 | 774 | 639 | | 7764 |
|  | lower extremity | 947 | 980 | 921 | 666 | 522 | 424 | 337 | 365 | | 5164 |
|  | Other | 168 | 194 | 195 | 181 | 235 | 321 | 339 | 489 | | 2121 |
|  | Total | 3198 | 3882 | 4232 | 3563 | 3558 | 4084 | 4056 | 4859 | | 31430 |
| **M&F** | Spine | 237 | 303 | 342 | 375 | 420 | 519 | 480 | 440 | | 3115 |
|  | Hip | 253 | 403 | 571 | 620 | 891 | 1484 | 1895 | 2773 | | 8889 |
|  | upper extremity | 1380 | 1418 | 1483 | 1099 | 983 | 949 | 769 | 710 | | 8789 |
|  | Wrist/ distal forearm | 990 | 1326 | 1468 | 1208 | 1059 | 1031 | 822 | 671 | | 8574 |
|  | lower extremity | 1330 | 1302 | 1199 | 842 | 661 | 521 | 406 | 410 | | 6670 |
|  | Other | 259 | 282 | 281 | 244 | 289 | 370 | 380 | 525 | | 2629 |
|  | Total | 4448 | 5034 | 5345 | 4386 | 4301 | 4874 | 4752 | 5528 | | 38667 |

25

**Table A3. Incidence of osteoporosis-related fractures (per 100,000 person years), by gender, fracture type and age-category, in 2010**

|  |  | **Osteoporosis-related fractures (/100,000)** | | | | | | | | | |
| --- | --- | --- | --- | --- | --- | --- | --- | --- | --- | --- | --- |
|  | **Fracture type** | **51-55** | **56-60** | **61-65** | **66-70** | **71-75** | **76-80** | **81-85** | **85+** | **total** | |
| **Male** | spine | 14 | 18 | 21 | 24 | 34 | 53 | 71 | 80 | | 27 |
|  | hip | 16 | 25 | 34 | 48 | 77 | 151 | 263 | 490 | | 69 |
|  | upper extremity | 74 | 65 | 57 | 51 | 49 | 62 | 70 | 87 | | 62 |
|  | Wrist/ distal forearm | 27 | 28 | 28 | 30 | 30 | 32 | 36 | 38 | | 29 |
|  | lower extremity | 65 | 59 | 52 | 46 | 47 | 45 | 52 | 52 | | 54 |
|  | Other | 15 | 16 | 16 | 16 | 18 | 23 | 30 | 42 | | 18 |
|  | Total | 212 | 212 | 207 | 215 | 255 | 367 | 521 | 789 | | 260 |
| **Female** | Spine | 27 | 38 | 43 | 72 | 98 | 144 | 177 | 176 | | 76 |
|  | Hip | 27 | 50 | 73 | 111 | 204 | 413 | 709 | 1112 | | 226 |
|  | upper extremity | 161 | 197 | 221 | 230 | 257 | 291 | 310 | 300 | | 228 |
|  | Wrist/ distal forearm | 141 | 218 | 247 | 278 | 298 | 343 | 355 | 302 | | 251 |
|  | lower extremity | 162 | 182 | 173 | 170 | 160 | 151 | 155 | 172 | | 167 |
|  | Other | 29 | 36 | 37 | 46 | 72 | 114 | 156 | 231 | | 69 |
|  | Total | 545 | 721 | 794 | 907 | 1089 | 1457 | 1862 | 2293 | | 1018 |
| **M&F** | Spine | 20 | 28 | 32 | 48 | 68 | 105 | 137 | 148 | | 53 |
|  | Hip | 21 | 37 | 53 | 80 | 144 | 299 | 540 | 935 | | 151 |
|  | upper extremity | 117 | 131 | 139 | 142 | 159 | 191 | 219 | 239 | | 150 |
|  | Wrist/ distal forearm | 84 | 122 | 137 | 156 | 171 | 208 | 234 | 226 | | 146 |
|  | lower extremity | 113 | 120 | 112 | 108 | 107 | 105 | 116 | 138 | | 114 |
|  | Other | 22 | 26 | 26 | 31 | 47 | 75 | 108 | 177 | | 45 |
|  | Total | 378 | 465 | 499 | 565 | 695 | 983 | 1353 | 1863 | | 659 |

26

**Table A4. Mean annual trend in incidence of hip fractures over 2000 to 2010 in both men & women (in %).**

|  | **Mean annual trend**  **over 2000-2010** | |
| --- | --- | --- |
|  | **Men** | **Women** |
| 50-55 years | 3.24% | 2.66% |
| 55-60 years | 8.59% | 6.60% |
| 60-65 years | 3.96% | 3.70% |
| 65-70 years | 3.25% | -0.05% |
| 70-75 years | 0.46% | -0.71% |
| 75-80 years | -0.84% | -0.92% |
| 80-87 years | 0.39% | -0.71% |
| >85 year years | 2.03% | 0.37% |
